# *Phytophthora capsici* genome assembly for two isolates using long-read Oxford Nanopore Technology sequencing

**DOI:** 10.1128/MRA.00196-23

**Published:** 2023-11-10

**Authors:** Emmanuel Szadkowski, Jacques Lagnel, Nasradin Touhami, Amalia Sayeh, Céline Lopez-Roques, Olivier Bouchez, Véronique Lefebvre

**Affiliations:** 1 INRAE, GAFL, Montfavet, France; 2 INRAE, GeT-PlaGe, Genotoul, Castanet-Tolosan, France; University of California, Riverside, Riverside, California, USA

**Keywords:** plant pathogens, oomycetes

## Abstract

The oomycete *Phytophthora capsici* is a common pathogen of the Solanaceae and Cucurbitaceae families. An improved assembly for the reference isolate LT1534 was constructed using Oxford Nanopore Technologies and Illumina data. Additionally, an unpolished assembly was produced for the European isolate Pc285 collected on chili pepper using Oxford Nanopore reads.

## ANNOUNCEMENT

*Phytophthora capsici* is a widespread pathogen that causes damages to many plant species, and particularly the species of the Solanaceae and Cucurbitaceae families. Several improved genomes using long-read technologies have recently been published for the cucurbit-infecting strain LT1534 (1), its original backcrossing recurrent parent LT263 (2), and several other chili pepper-infecting strains collected in China (3) and Korea (4). Here, we have produced long-read nanopore assemblies of the reference isolate LT1534 and provided an unpolished assembly of the isolate Pc285 that was sampled from chili pepper in France.

The two *P. capsici* isolates LT1534 (Pc331 in the INRAE GAFL collection) and Pc285 were grown on a clarified liquid V8 medium, supplemented with a cocktail of antibiotics, for 7 days in the dark, and DNA extraction was performed following the protocol of Panabières and Le Berre (5).

Library preparation and sequencing were performed at the INRAE GeT-PlaGe core facility, according to the manufacturer’s instructions, “1D gDNA selecting for long reads (SQK-LSK109).” At each step, DNA was quantified using the Qubit dsDNA HS Assay Kit (Life Technologies). DNA purity was tested using the NanoDrop (Thermo Fisher) and size distribution, and degradation was assessed using the Agilent Fragment Analyzer DNF-464 HS Large Fragment Kit. Purification steps were performed using AMPure XP beads (Beckman Coulter). For LT1534, 9 µg of DNA was purified, and a size selection step using a Short-Read Eliminator XS Kit (Circulomics) was performed. For Pc285, 5 µg of DNA was purified and then sheared at 35 kb using the Megaruptor system (Diagenode). For both prepared samples, a one-step DNA damage repair + END-repair + dA tail of double-stranded DNA fragments was performed on 2 µg of sample. Then, adapters were ligated to the libraries. Each library was loaded onto one R9.4.1 flow cell and sequenced on a GridION instrument (Gridion-release 18.12.4–1 and 19.06.9–1) at 27 fmol and 21 fmol, respectively, within 48 h. The base calling was performed by Guppy 2.0.10–1 and 3.0.6–1.

Sequencing produced 21.1 Gbp for LT1534 with 1.4 million reads and a median read length of 13.0 kbp. Around 17.5 Gbp was obtained for Pc285, representing 1.3 million reads with a median read length of 11.3 kbp. Nanopore reads were preprocessed with Porechop (https://github.com/rrwick/Porechop, version 0.2.4), followed for Pc285 by Filtlong read selection (QC >7, read length >3 kbp, https://github.com/rrwick/Filtlong, version 0.2.0). Canu version 1.8 (6) correction was applied, and assembly was performed using SMARTdenovo version 1.11 (7). Contigs of LT1534 were further polished in two passes with Pilon version 1.23 (8) using LT1534 Illumina single-ended reads published by Lamour et al. (9). The total length of the assembly was smaller for Pc285 (75.7 Mbp with 209.0 kbp contig median length) than for LT1534 (92.6 Mbp with 347.6 kbp contig median length), with values comparable to other genome assemblies (Table 1). The assembly resulted in an N50 length of 715 kbp and 610 kbp, and 50% of the assembly is contained in 41 and 39 contigs for LT1534 and Pc285, respectively.

**TABLE 1:** Overview of the recent *Phytophthora capsici* genome assembly metrics

Article	Lee et al. (4)			Shi et al. (3)	Stajich et al. (1)	This article	
Isolate	JHAI1-7	KPC-7	MY-1	SD33	LT1534	LT1534	Pc285
Assembly statistics							
Total number of contigs	472	548	521	194	782	203	213
Total length (bp)	76,839,304	74,832,015	76,624,583	100,526,817	94,176,027	92,550,228	75,652,381
Average length (bp)	162,795	136,555	147,072	518,179	120,430	455,912	355,176
Median length (bp)	29,583	26,079	26,730	296,807	26,215	347,626	209,050
Min length (bp)	1,229	657	723	11,508	2,527	16,903	39,489
Max length (bp)	2,514,355	2,715,842	2,720,928	4,147,862	4,616,793	2,689,262	2,757,130
Total Ns	1,067	1,304	1,409	0	100	0	0
Total %GC	51.01	50.96	50.96	50.82	51.19	50.94	50.8
N50 length (bp)	798,104	663,153	635,553	1,013,378	485,876	714,920	609,692
Busco protist_ensembl (*n* = 215)							
Complete	99.5%	99.0%	100.0%	95.4%	99.0%	97.2%	78.6%
Single	95.3%	98.1%	98.6%	72.1%	82.3%	76.2%	67.9%
Duplicated	4.2%	0.9%	1.4%	23.3%	16.7%	20.5%	10.7%
False	0.0%	0.0%	0.0%	0.5%	0.0%	0.0%	2.8%
Missing	0.5%	1.0%	0.0%	4.1%	1.0%	2.8%	18.6%

Busco (version 3.0.2) score was assessed for the LT1534 assembly on the “protist_ensembl” data set, achieving 97.2% completeness out of 215 genes (76.2% unique, 20.5% duplicated, 0% fragmented, and 2.8% missing) in the same range of other papers (Table 1). Given the unpolished nature of the Pc285 assembly, its Busco score is lower with 78.6% of completeness (Table 1).

LT1534 annotation was performed using Funannotate 1.8.14 with default parameters (10). Briefly, gene prediction of repeat-masked contigs (using tantan) was performed using RefSeq proteins from the NCBI database (filtered on txid4783) and without RNA-Seq experimental data, producing 23,347 predicted genes including 2,100 genes coding tRNAs. Functional annotation was completed using InterProScan (version 5.42–78.0).

## Data Availability

Raw data, assemblies, and annotations for isolates LT1534 and Pc285 are available in Project PRJEB60432 of the ENA repository.
